# Visual Snow Syndrome Improves With Modulation of Resting-State Functional MRI Connectivity After Mindfulness-Based Cognitive Therapy: An Open-Label Feasibility Study

**DOI:** 10.1097/WNO.0000000000002013

**Published:** 2023-11-15

**Authors:** Sui H. Wong, Giuseppe Pontillo, Baris Kanber, Ferran Prados, Janet Wingrove, Marios Yiannakas, Indran Davagnanam, Claudia A. M. Gandini Wheeler-Kingshott, Ahmed T. Toosy

**Affiliations:** Guy's and St Thomas' NHS Foundation Trust (SHW), London, United Kingdom; Department of Neuroinflammation (GP, BK, FP, MY, ID, CAMGW-K, ATT), UCL Queen Square Institute of Neurology, University College London, London, United Kingdom; Moorfields Eye Hospital (SHW, ID, ATT), London, United Kingdom; King's College London (SHW), London, United Kingdom; South London and Maudsley NHS Foundation Trust (JW), London, United Kingdom; Centre for Medical Image Computing (BK, FP), University College London, London, United Kingdom; NIHR Biomedical Research Centre at UCLH and UCL (BK, FP), London, United Kingdom; Department of Brain & Behavioural Sciences (CAMGW-K), University of Pavia, Pavia, Italy; Brain Connectivity Center (CAMGW-K), IRCCS Mondino Foundation, Pavia, Italy; and Department of Clinical and Movement Neurosciences (SHW), UCL Queen Square Institute of Neurology, University College London, London, United Kingdom.

## Abstract

**Background:**

Visual snow syndrome (VSS) is associated with functional connectivity (FC) dysregulation of visual networks (VNs). We hypothesized that mindfulness-based cognitive therapy, customized for visual symptoms (MBCT-vision), can treat VSS and modulate dysfunctional VNs.

**Methods:**

An open-label feasibility study for an 8-week MBCT-vision treatment program was conducted. Primary (symptom severity; impact on daily life) and secondary (WHO-5; CORE-10) outcomes at Week 9 and Week 20 were compared with baseline. Secondary MRI outcomes in a subcohort compared resting-state functional and diffusion MRI between baseline and Week 20.

**Results:**

Twenty-one participants (14 male participants, median 30 years, range 22–56 years) recruited from January 2020 to October 2021. Two (9.5%) dropped out. Self-rated symptom severity (0–10) improved: baseline (median [interquartile range (IQR)] 7 [6–8]) vs Week 9 (5.5 [3–7], *P* = 0.015) and Week 20 (4 [3–6], *P* < 0.001), respectively. Self-rated impact of symptoms on daily life (0–10) improved: baseline (6 [5–8]) vs Week 9 (4 [2–5], *P* = 0.003) and Week 20 (2 [1–3], *P* < 0.001), respectively. WHO-5 Wellbeing (0–100) improved: baseline (median [IQR] 52 [36–56]) vs Week 9 (median 64 [47–80], *P* = 0.001) and Week 20 (68 [48–76], *P* < 0.001), respectively. CORE-10 Distress (0–40) improved: baseline (15 [12–20]) vs Week 9 (12.5 [11–16.5], *P* = 0.003) and Week 20 (11 [10–14], *P* = 0.003), respectively. Within-subject fMRI analysis found reductions between baseline and Week 20, within VN-related FC in the i) left lateral occipital cortex (size = 82 mL, familywise error [FWE]-corrected *P* value = 0.006) and ii) left cerebellar lobules VIIb/VIII (size = 65 mL, FWE-corrected *P* value = 0.02), and increases within VN-related FC in the precuneus/posterior cingulate cortex (size = 69 mL, cluster-level FWE-corrected *P* value = 0.02).

**Conclusions:**

MBCT-vision was a feasible treatment for VSS, improved symptoms and modulated FC of VNs. This study also showed proof-of-concept for intensive mindfulness interventions in the treatment of neurological conditions.

## Abstract 2

Supplemental Digital Content is Available in the Text.

Visual snow syndrome (VSS) is a neurological syndrome associated with functional dysregulation of vision-related brain networks.^1^ Treatments are limited, and no studies have studied treatment-associated brain functional modifications.

We report an open-label feasibility intervention study that includes resting-state (rs)-fMRI as an objective outcome measure. We hypothesized that mindfulness-based cognitive therapy customized for visual symptoms (MBCT-vision) can treat VSS and modulate functional dysregulation in vision-related pathways.

Mindfulness is the nonjudgmental awareness of the present moment. Studies on mindfulness-based interventions have shown changes in neural networks and improved psychological resilience.^2^ The clinical application of mindfulness is based on the training of attention, awareness, and self-regulation.

MBCT is a clinical intervention related to mindfulness-based stress reduction and cognitive-behavioral therapy (CBT), originally for major depression.^3^ MBCT is an 8-week program that develops skills of mindfulness and CBT strategies through weekly small-group participation and structured daily practice between sessions. MBCT trains attention, awareness, and emotional regulation, while embodying nonjudgment, curiosity, kindness, and friendliness.^3^ The facilitated group discussions on mindfulness practices are designed to develop cognitive-behavioral skills.

## METHODS

We performed a prospective open-label feasibility study of VSS, defined by the International Classification of Headache Disorders-3 diagnostic criteria. All participants were screened by the principal investigator (PI), a neuro-ophthalmologist (S.H.W.) before study recruitment.

Participants were enrolled into an 8-week program of MBCT-vision delivered by an experienced MBCT teacher (J.W.) and assisted by the PI and an MBCT teacher (S.H.W.). The standard MBCT protocol was customized to the MBCT-vision protocol by replacing structured discussions about preventing depression with structured discussions related to the impact of and response to visual symptoms.^3^ Group sizes were limited to facilitate effective group learning and engagement. The MBCT-vision intervention was delivered in 3 groups to enable sufficient study sample size. The first group met in-person; the second and third groups met online because of the COVID-19 pandemic.

Primary outcome measures were changes in self-reported visual symptom severity and impact of symptoms on daily life, comparing scores at baseline, Week 9 (1 week after MBCT-vision completion), and Week 20 (i.e., 3 months after MBCT-vision intervention to evaluate sustained treatment response). The self-reported ratings were determined from an ordinal scale with a range of 0–10, where 0 indicated no symptoms or impact, and 10 indicated maximum severity or impact. Statistical analysis used nonparametric 2-tailed Wilcoxon signed-rank tests, with a nominal *P* value of significance of 0.0182,^4^ resulting in an overall significance level of 0.05.

Secondary outcome measures were the overall WHO-5 Wellbeing questionnaire score measured on a scale of 0–100^5^ and the overall CORE-10 Psychological Distress questionnaire score measured on a scale of 0–40,^6^ comparing baseline with Week 9 and Week 20. Statistical analyses for these primary and secondary outcomes used nonparametric 2-tailed Wilcoxon signed-rank tests, with a nominal *P* value of significance of 0.0182,^4^ resulting in an overall significance level of 0.05.

Quantitative MRI was performed for the second and third groups after funding was obtained after an interim report on the first group.^7^ Secondary MRI outcomes included resting-state functional connectivity and diffusion MRI microstructural metrics compared between baseline and Week 20. Healthy control MRI data were compared with VSS to investigate fMRI and diffusion-related alterations at baseline only.

### MRI Data Acquisition and Analysis

MRI was performed on a 3T Philips Ingenia CX system (Philips Healthcare, Best, the Netherlands), with a brain protocol including structural T1-weighted (T1w) volume acquired using a 3D magnetization prepared turbo field-echo sequence with repetition time (TR) = 6.9 milliseconds; echo-time (TE) = 3.2 milliseconds; inversion delay time = 823 milliseconds; flip angle = 8°; voxel size = 1 × 1 × 1 mm^3^; and compressed-SENSE acceleration factor = 6, which was used as anatomical reference; a 3D fluid-attenuated inversion recovery (FLAIR) volume was acquired to screen for incidental pathology (TR = 4800 milliseconds; TE = 264 milliseconds; TI = 1650 milliseconds; and voxel size = 1 × 1 × 1 mm^3^). T2*-weighted volumes were acquired using a gradient‐echo echo‐planar imaging sequence (TR = 4000 milliseconds; TE = 25 milliseconds; voxel size = 2.9 × 2.9 × 3.5 mm^3^; and 200 time points were acquired in 2 runs of 100), for functional connectivity (FC) analysis. Diffusion-weighted imaging (DWI) volumes (b values 0, 1,000, 2,000, and 2,800 s/mm^2^; number of volumes: 83; voxel size = 2 × 2 × 2 mm^3^; TR = 6078 milliseconds; and TE = 96 milliseconds) for diffusion tensor imaging (DTI) and neurite orientation dispersion and density imaging (NODDI) analyses. A separate reversed phase encoding volume, using identical acquisition parameters as the main DWI scan, was acquired to correct for echo planar imaging distortions.

Preprocessing of rs-fMRI data was performed using fMRIPrep 20.2.6^8^ and included correction for head motion, susceptibility distortions and slice-timing, registration to the T1w volumes, and denoising to minimize residual non-neuronal variability (See **Supplemental Digital Content**, **Supplementary Material**, http://links.lww.com/WNO/A781). Preprocessed fMRI data were imported into the functional connectivity (CONN) toolbox (v.21.b, http://www.nitrc.org/projects/conn) for the subsequent analyses. We used CONN's independent component analysis (ICA) implementation (including temporal concatenation across multiple subjects, group-level dimensionality reduction using principal component analysis, FastICA to obtain 10 spatially independent components, and subject-specific back projection using dual regression)^9^ to identify the visual network (VN). Subject-level VN maps entered second-level analyses, where we tested in a general linear model framework: (i) within-subject modifications of FC before and after MBCT-vision, (ii) associations between pre–post FC changes and clinical improvement (defined as a self-rated symptom severity score delta >2), and (iii) cross-sectional differences between VSS patients at baseline and healthy controls (HCs). For all analyses, significance level was set at α = 0.05, familywise error (FWE)-corrected at cluster level.

DTI and NODDI models were fitted to the DWI data, after eddy current, distortion, and motion correction, using FSL^10^ and the MATLAB (MathWorks, Inc, Natick, MA) NODDI toolboxes (http://nitrc.org/projects/noddi_toolbox). We assessed voxelwise differences in core white matter voxels of DTI (fractional anisotropy and mean diffusivity) and NODDI (neurite density index, orientation dispersion index, and isotropic volume fraction) metrics (i) between VSS patients at baseline and HCs and (ii) within VSS patients before and after intervention using the tract-based spatial statistics (TBSS) toolbox.^11^ Threshold-free cluster enhancement was used, and FWE-corrected *P* values less than 0.05 were considered statistically significant.

This study received ethics approval from the National Research Ethics Committee (London-South East, 19/LO/1615) and registered on ClinicalTrials.gov (Identifier: NCT04184726). Written informed consent was obtained from all participants, and the appropriate institutional forms have been archived.

### Data Availability

Anonymized data beginning 12 months and ending 3 years after publication of this article can be made available on request from a qualified researchers whose proposed use of the data is approved by the PI and the institution's research governance committee, and requesters will need to sign a data access agreement.

## RESULTS

Twenty-one participants (14 male participants, median age 30 years, range 22–56 years) with VSS were recruited from January 2020 to October 2021. Two participants (9.5%) did not complete the 8-week program citing work-related reasons and dropped out of the study. Pre-existing migraine was present in 12/21 (57%) and anxiety in 9/21(43%).

**FIG. 1.**
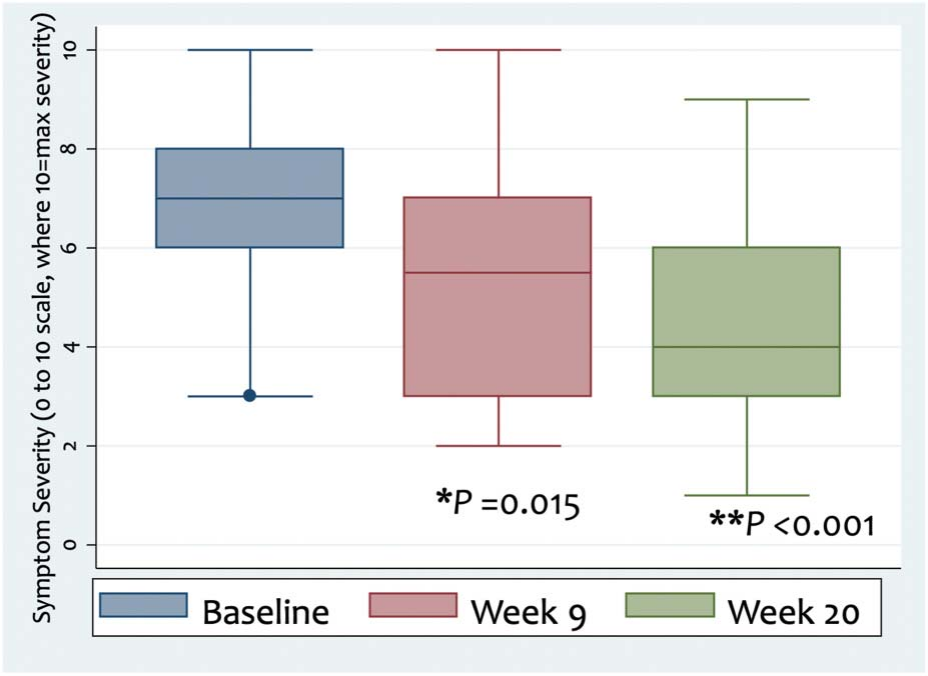
Self-rated symptom severity on a 0–10 ordinal scale at baseline (median 7, IQR 6–8), Week 9 (median 5.5, IQR 3–7, *P = 0.015), and Week 20 (median 4, IQR 3–6, **P < 0.001) of study entry, showing significant improvement after the 8-week MBCT-vision program. MBCT indicates mindfulness-based cognitive therapy; IQR, interquartile range.

Self-rated symptom severity (0–10 scale) improved (Fig. 1), from baseline (median 7, interquartile range [IQR] 6–8) to Week 9 (median 5.5, IQR 3–7; median change −2 with 95% confidence interval [95% CI] −3 to 0, *P* = 0.015) and Week 20 (median 4, IQR 3–6; median change −3 with 95% CI −4 to −1, *P* <0.001), respectively, where a more negative score indicates lower severity.

**FIG. 2.**
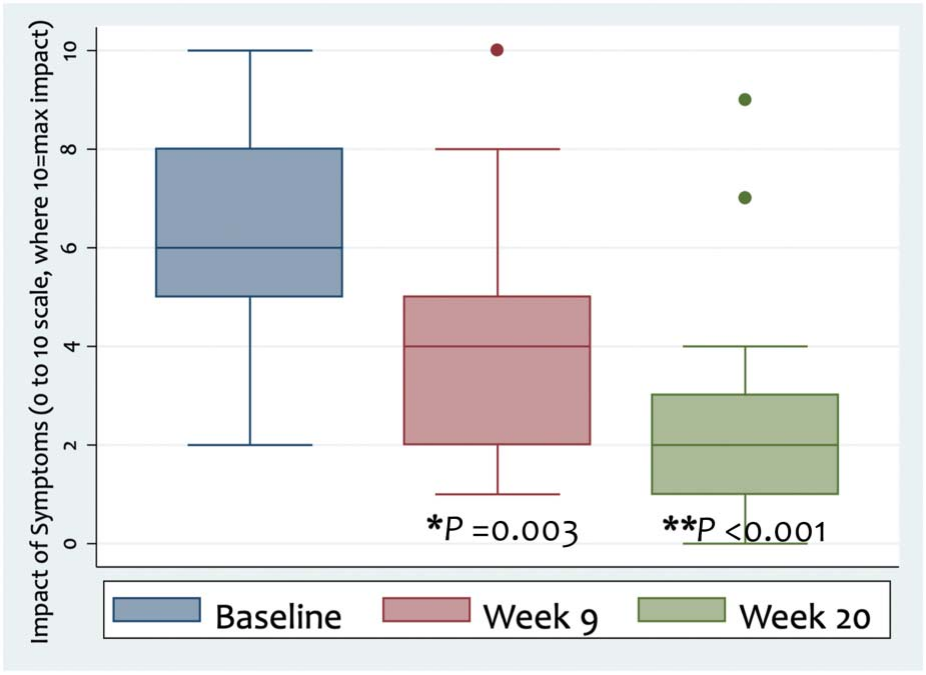
Self-rated impact of symptoms on daily life on a 0–10 ordinal scale, at baseline (median 6, IQR 5–8), Week 9 (median 4, IQR 2–5, *P = 0.003), and Week 20 (median 2, IQR 1–3, **P < 0.001) of study entry, showing significant improvement after the 8-week MBCT-vision program. MBCT indicates mindfulness-based cognitive therapy; IQR, interquartile range.

Self-rated impact of symptoms on daily life (0–10 scale) improved (Fig. 2) from baseline (median 6, IQR 5–8), compared with Week 9 (median 4, IQR 2–5; median change −3 with 95% CI −3.7 to −1, *P* = 0.003), and Week 20 (median 2, IQR 1–3; median change −4 with 95% CI −5 to −1.7, *P* < 0.001), where a more negative score indicates lower impact of symptoms on daily life.

Post hoc analyses compared those with and without pre-existing migraine and anxiety, respectively, finding no significant differences in pre-treatment and post-treatment changes for the primary outcomes (See **Supplemental Digital Content**, **Table 1**, http://links.lww.com/WNO/A781).

WHO-5 Wellbeing scores (range 0–100) improved from baseline (median 52, IQR 36–56) to Week 9 (median 64, IQR 47–80; median change +16 with 95% CI 6.3–22.8; *P* = 0.001) and Week 20 (median 68, IQR 48–76; median change *+*16 with 95% CI 8–24*; P* < 0.001), where an increase in scores indicated an improvement in wellbeing.

CORE-10 Psychological Distress scores (0–40 scale) improved from baseline (median 15, IQR 12–20) to Week 9 (median 12.5, IQR 11–16.5; median change −3 with 95% CI −5 to 0; *P* = 0.003) and Week 20 (median 11, IQR 10–14; median change −3 with 95% CI −6 to −2; *P* = 0.003), respectively, where scores for categories of distress are as follows: healthy (0–5), low (6–10), mild (11–14), moderate (15–19), moderate‐to‐severe (20–24), and severe (25 and above),^12^ and a more negative score indicates less distress.

Of the 14 VSS patients (9 M, median age 29 years, IQR 24.5–32.5) undergoing brain MRI at baseline, one dropped out of the study and was not scanned at Week 20. Sixteen HCs who were age-matched (mean age 34.9 years, SD 7.2, *P* = 0.06, Student *t* test) and sex-matched (8 M, *P* = 0.43, Pearson χ^2^ test) were imaged cross-sectionally as a baseline control group.

**FIG. 3.**
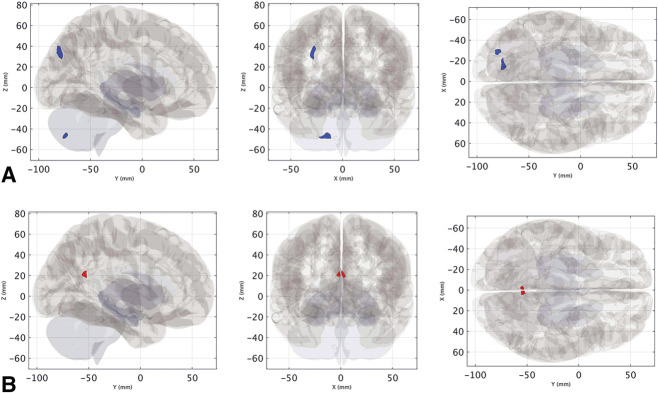
Clusters of significantly reduced (**A**) and increased (**B**) VN-related FC over time, displayed as binary masks (i.e., thresholded and binarized statistical maps) on a 3D glass brain representation. FC indicates functional connectivity; VN, visual network.

The within-VSS-subject (baseline vs Week 20 or pre–MBCT-vision vs post–MBCT-vision treatment, N = 13) fMRI analysis revealed reductions over time in VN-related FC within the left lateral occipital cortex (cluster size = 82 mL, cluster-level FWE-corrected *P* value = 0.006) and the left cerebellar lobules VIIb/VIII (size = 65 mL, cluster-level FWE-corrected *P* value = 0.02) (Fig. 3A, See **Supplemental Digital Content**, **Table 1**, http://links.lww.com/WNO/A781), along with increases in FC within the precuneus/posterior cingulate cortex (cluster size = 69 mL, cluster-level FWE-corrected *P* value=0.02) (Fig. 3B, See **Supplemental Digital Content**, **Table 1**, http://links.lww.com/WNO/A781) post–MBCT-vision (Week 20) compared with pre–MBCT-vision (baseline).

**FIG. 4.**
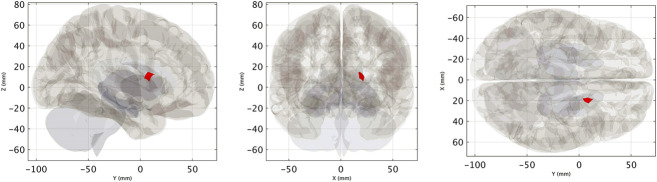
Cluster of significant association between pre–post VN-related FC increase and clinical improvement (defined as a self-rated symptom severity score delta >2), displayed as a binary mask (i.e., thresholded and binarized statistical map) on a 3D glass brain representation (See **Supplemental Digital Content, Table 3**, http://links.lww.com/WNO/A781). FC indicates functional connectivity; VN, visual network.

**FIG. 5.**
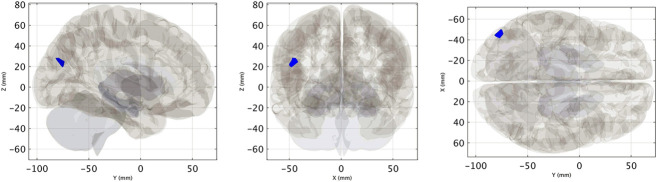
Cluster of significant between-group (VSS < HC) difference in terms of VN-related FC, displayed as a binary mask (i.e., thresholded and binarized statistical map) on a 3D glass brain representation. FC indicates functional connectivity; HC, healthy controls; VN, visual network; VSS, visual snow syndrome.

There was a significant association between the improvement in self-reported severity score and increased VN-related FC within the right striatum (size = 70 mL, cluster-level FWE-corrected *P* value = 0.03) (Fig. 4, See **Supplemental Digital Content**, **Table 2**, http://links.lww.com/WNO/A781). For the between-group analysis (VSS vs HC, N = 13 vs 16), VSS subjects at baseline showed reduced VN-related FC within the left lateral occipital cortex (cluster size = 118 mL, cluster-level FWE-corrected *P* value = 0.01) compared with HCs (Fig. 5, See **Supplemental Digital Content**, **Table 4**, http://links.lww.com/WNO/A781). No significant differences were found for DTI (fractional isotropy and mean diffusivity) and NODDI (neurite density index, orientation dispersion index, and isotropic volume fraction) metrics between VSS patients at baseline and controls and VSS patients before and after intervention.

## DISCUSSION

There are no other previous intervention study on VSS with rs-fMRI as a neural outcome correlate, confirmed on a PubMed search. We show MBCT-vision is a feasible treatment for VSS, with subjective improvements and fMRI-associated changes.

Subjective improvements in symptom severity and impact of symptoms were sustained at three months after intervention. Although there was no treatment control group for comparison because of the study's open-label feasibility design, such a sustained improvement is unexpected from natural history studies, showing that VSS persists without spontaneous improvement.^13^

Our primary outcomes were an ordinal scale (0–10) for the rating of the severity of VSS and the impact of this on daily life. This was a pragmatic study design because of the lack of validated rating scales for VSS. Participants were advised to respond based on all aspects of their VSS. Future studies validating a VSS rating scale that differentiates all aspects of VSS, including the primary symptom of visual snow and other visual symptoms such as after-images and floaters, are necessary to advance research in VSS.

We showed objective fMRI reorganization of the visual network, involving both visual and extravisual areas in the neocortex and cerebellum, after MBCT-vision intervention. Alterations of FC in VSS have previously involved both intranetwork dysconnectivity of vision-related brain regions and aberrant coupling with other functional systems, including the default mode network (DMN) and the thalami/basal ganglia.^14^

Our findings of reduced VN-related FC in the extrastriate visual cortex in VSS patients compared with HC are consistent with this. Moreover, we found that MBCT-vision intervention was associated with functional reorganization of the VN within the visual extrastriate regions of the lateral occipital cortex, the posterior cerebellar areas implicated in visual processing and attentional mechanisms,^15^ and the posterior hub of the DMN. These findings support the hypothesis that the alteration of VN-related FC within visual areas and association networks may underpin disruptions between the integration of internally generated visual information and the processing of salient environmental stimuli, resulting in a constant “noise-like” perception.^14^

The modulation of FC within the right striatum was related to clinical improvement. There is evidence that the basal ganglia influence visual perception, although the visual corticostriatal loop^16^ and altered functional coupling between visual hubs and the caudate nuclei have also been reported with VSS.^14^ We therefore speculate that MBCT-vision improved visual snow symptoms by modulating FC between the VN and the striatum.

There is emerging understanding of the role of the cerebellum in processing not only motor functions, but also cognitive and associative functions.^17^ Of note, MBCT-vision intervention modulated functional connectivity of the VIIb/VIII cerebellar lobule. Considering that the cerebellum has a role in prediction and somatosensory state estimation as well as in cognitive control,^18^ it is possible that the MBCT-vision intervention could have modulated the response of the cerebellum to visual stimuli. The cerebellum also impacts on brain dynamics, at whole-brain network level.^19^ Therefore, one could hypothesize that changes in lobule VIIb/VIII may have driven visual network changes. Future research in advanced signal analysis, such as dynamic causal modeling of resting-state data, will be of interest to clarify how MBCT-vision acts on different cortical areas.

Mindfulness-based approaches such as MBCT empower patients with skillsets that enable self-efficacy for the management of their conditions.^3^ This may explain the significant reduction of the impact of symptoms on daily life of any ongoing symptoms after MBCT-vision intervention. In addition, participants had significant improvements in the WHO-5 Wellbeing Index^5^ and the CORE-10 Psychological Distress scale.^6^ In comparison, the natural history of VSS is associated with unchanged levels of anxiety and depression.^13^

Our study showed MBCT-vision intervention resulted in changes to the visual and its associated default mode networks using fMRI. The neurological network dysfunction of VSS affects the visual, attentional, and salience networks.^1^ The salience network and default mode networks are closely associated, where the default mode network reflects the baseline state of connectivity. Mindfulness meditations have been shown to modulate these networks.^2^ This observation of change in the default mode network was also seen in a similar study on tinnitus, which is often associated with VSS, whereby MBCT intervention resulted in changes of the default mode network on fMRI.^20^ In line with this, a systematic review of mindfulness-based interventions on tinnitus showed a decrease in tinnitus distress scores.^21^

A possible conclusion is that MBCT-vision improves VSS symptoms but not the underlying condition itself. However, a strength of our study is the inclusion of objective fMRI measures, which showed modulation of visual network areas, suggesting a material change in the underlying condition.

Limitations of this study include the lack of a treatment control group. This will be addressed in an upcoming randomized controlled trial (RCT) which has been made possible after this feasibility study (ClinicalTrials.gov identifier: NCT06018103, www.MBCT-vision.co.uk). Another limitation is the lack of detailed evaluation of nonvisual symptoms, e.g., auditory, vestibular, and somaesthetic symptoms; this is also planned in the upcoming RCT.

### Conclusions

MBCT-vision is a potential treatment for VSS, with benefits that translate to quality of life, self-efficacy, and wellbeing. This feasibility study supports the rationale for a randomized controlled trial that includes MBCT-vision as a treatment arm (ClinicalTrials.gov identifier: NCT06018103, www.MBCT-vision.co.uk). Our study showed that mindfulness intervention can change organization of functional networks on fMRI, an objective observation raising the possibility of mindfulness therapy in the treatment of other neurological conditions.

STATEMENT OF AUTHORSHIP

Conception and design: S. H. Wong, G. Pontillo, B. Kanber, F. Prados, J. Wingrove, C. A. M. Gandini Wheeler-Kingshott, A. T. Toosy; Acquisition of data: S. H. Wong, J. Wingrove, M. Yiannakas, A. T. Toosy; Analysis and interpretation of data: S. H. Wong, G. Pontillo, B. Kanber, F. Prados, J. Wingrove, I. Davagnanam, C. A. M. Gandini Wheeler-Kingshott, A. T. Toosy. Drafting the manuscript: S. H. Wong, G. Pontillo, A. T. Toosy; Revising the manuscript for intellectual content: S. H. Wong, G. Pontillo, B. Kanber, F. Prados, J. Wingrove, M. Yiannakas, I. Davagnanam, C. A. M. Gandini Wheeler-Kingshott, A. T. Toosy. Final approval of the completed manuscript: S. H. Wong, G. Pontillo, B. Kanber, F. Prados, J. Wingrove, M. Yiannakas, I. Davagnanam, C. A. M. Gandini Wheeler-Kingshott, A. T. Toosy.

## Supplementary Material

SUPPLEMENTARY MATERIAL
