# Supplementary material for: Visual Snow Syndrome Improves With Modulation of Resting-State Functional MRI Connectivity After Mindfulness-Based Cognitive Therapy: An Open-Label Feasibility Study
Source: J Neuroophthalmol. 2023 Nov 15;44(1):112–8. doi: 10.1097/WNO.0000000000002013 (PMC10855987; doi:10.1097/WNO.0000000000002013)
Supplement: SUPPLEMENTARY MATERIAL [file jno-44-112-s001.docx]

**MBCT-vision Supplementary File**

**Supplementary Table 1**

Legend: Post-hoc analysis of the change in primary outcome measures, showing median change in score with IQR limits. Analysis by 2-tailed Wilcoxon Signed Ranks Test. The negative numbers indicate a reduction in (1) severity of symptoms or (2) impact of symptoms on daily life, as rated by participants on a 0 to 10 ordinal scale, where 10 indicated maximum severity or impact respectively.

|  |  | Migraine | | | Anxiety | | |
| --- | --- | --- | --- | --- | --- | --- | --- |
|  |  | Yes | No | p-value | Yes | No | p-value |
| 1. Severity of Symptoms | Week-9 compared to Baseline | -1.5 (-4.0 to 0.0) | -2.0 (-3.0 to 1.75) | 0.622 | -3.0 (-2.0 to -2.5) | -1.0 (-4.0 to 0.25) | 0.538 |
|  | Week-20 compared to Baseline | -3.0 (-4.75 to -1.25) | -2.5 (-3.25 to 1.25) | 0.136 | -5.0 (-4.0   to -1.0) | -2.0 (-4.5 to -1.75) | 0.718 |
| 1. Impact of Symptoms on Daily Life | Week-9 compared to Baseline | -3.0 (-4.0 to -1.5) | -1 .0 (-3.25 to 0.75) | 0.216 | -3.0 (-3.75 to -1.0) | -3.0 ( -4.0 to 0.0) | >0.999 |
|  | Week-20 compared to Baseline | -5.0 (-5.0  to -1.75) | -2.0 (-4.5 to -1.75) | 0.259 | -5.0 ( - 5.0 to -2.0) | -4.0 ( -5.25 to -1.0) | 0.549 |

**Supplementary Table 2**

**Table Legend:** Results of the within-subjects (pre- vs post-MBCT) fMRI analysis. Anatomical labelling is made according to the Harvard-Oxford (https://pubmed.ncbi.nlm.nih.gov/16530430/) and AAL (for the cerebellum)( https://pubmed.ncbi.nlm.nih.gov/11771995/) atlases.

| **Anatomic region** | **Size (mL)** | **MNI coordinates (mm)** | | | **p-value**  **(FWE-corrected)** |
| --- | --- | --- | --- | --- | --- |
|  |  | **x** | **y** | **z** |  |
| Pre > Post | | | | | |
| L sLOC | 82 | -32 | -82 | +32 | 0.006 |
| L Cerebellum VIIb/VIII | 65 | -12 | -74 | -44 | 0.02 |
| Pre < Post | | | | | |
| Precuneous / PCC | 69 | +2 | -52 | +18 | 0.02 |

**Supplementary Table 3**

**Table Legend:** Results of the fMRI analysis testing the association between pre-post increase of VN-related FC and clinical improvement (defined as a self-rated symptom severity score delta > 2). Anatomical labelling is made according to the Harvard-Oxford atlas (https://pubmed.ncbi.nlm.nih.gov/16530430/).

| **Anatomic region** | **Size (mL)** | **MNI coordinates (mm)** | | | **p-value**  **(FWE-corrected)** |
| --- | --- | --- | --- | --- | --- |
|  |  | **x** | **y** | **z** |  |
| R Putamen/Caudate | 70 | +20 | +08 | +12 | 0.03 |

**Supplementary Table 4**

**Table Legend:** Results of VSS < HC contrast at the cross-sectional between-group comparison in terms of VN-related FC. No significant results emerged for the VSS > HC contrast. Anatomical labelling is made according to the Harvard-Oxford atlas (https://pubmed.ncbi.nlm.nih.gov/16530430/).

| **Anatomic region** | **Size (mL)** | **MNI coordinates (mm)** | | | **p-value**  **(FWE-corrected)** |
| --- | --- | --- | --- | --- | --- |
|  |  | **x** | **y** | **z** |  |
| L sLOC | 118 | -48 | -80 | +28 | 0.01 |
